# CD47‐SIRPα blocking‐based immunotherapy: Current and prospective therapeutic strategies

**DOI:** 10.1002/ctm2.943

**Published:** 2022-07-31

**Authors:** Renée Bouwstra, Tom van Meerten, Edwin Bremer

**Affiliations:** ^1^ Department of Hematology University Medical Center Groningen University of Groningen Groningen The Netherlands

**Keywords:** bispecific antibody, CD47, immunotherapy, patient selection, tumour selective

## Abstract

**Background:**

The CD47‐signal regulatory protein alpha (SIRPα) ‘don't eat me’ signalling axis is perhaps the most prominent innate immune checkpoint to date. However, from initial clinical trials, it is evident that monotherapy with CD47‐SIRPα blocking has a limited therapeutic effect at the maximum tolerated dose. Furthermore, treatment is associated with severe side effects, most notably anaemia, that are attributable to the ubiquitous expression of CD47. Nevertheless, promising clinical responses have been reported upon combination with the tumour‐targeting antibody rituximab or azacytidine, although toxicity issues still hamper clinical application.

**Main body:**

Here, we discuss the current state of CD47‐SIRPα blocking therapy with a focus on limitations of current strategies, such as depletion of red blood cells. Subsequently, we focus on innovations designed to overcome these limitations. These include novel antibody formats designed to selectively target CD47 on tumour cells as well as tumour‐targeted bispecific antibodies with improved selectivity. In addition, the rationale and outcome of combinatorial approaches to improve the therapeutic effect of CD47 blockade are discussed. Such combinations include those with tumour‐targeted opsonizing antibodies, systemic therapy, epigenetic drugs, other immunomodulatory T‐cell‐targeted therapeutics or dual immunomodulatory CD47 bispecific antibodies.

**Conclusion:**

With these advances in the design of CD47‐SIRPα‐targeting therapeutic strategies and increasing insight into the mechanism of action of this innate checkpoint, including the role of adaptive immunity, further advances in the clinical application of this checkpoint can be anticipated.

## Graphical abstract

- CD47‐SIRPα blocking has met with several challenges in clinical application including lack of therapeutic effect and toxicity.
- Novel (bispecific) formats can increase tumor‐selectivity and reduced toxicity of CD47‐SIRPα‐based immunotherapy.
- Combination strategies improve efficacy of CD47‐SIRPα‐based immunotherapy.
- Increasing insight into the mechanism‐of‐action, including the role of adaptive immunity and timing can bring further advances in clinical application.

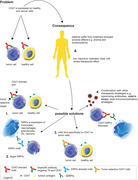

## 1 BACKGROUND

Phagocytes are the first line of defence in immunosurveillance and act upon the balance between ‘eat me’’ and ‘don't eat me’ signals to decide to phagocytose or not. Within this balance, the CD47‐SIRPα axis has emerged as a key player. CD47 is a 50 kDa penta‐spanning transmembrane protein belonging to the immunoglobulin family that acts as a ‘don't eat me’ signal.^1^ Upon binding of CD47 to signal regulatory protein alpha (SIRPα) expressed on phagocytes, immunoreceptor tyrosine‐based inhibitory motif (ITIM)‐mediated inhibitory signalling is initiated to block phagocytic removal. This signalling axis is, for instance, pivotal for red blood cell (RBC) homeostasis, with young RBCs expressing high levels of CD47 and a decrease in CD47 expression in ageing RBCs enabling effective removal.^2^

**FIGURE 1:**
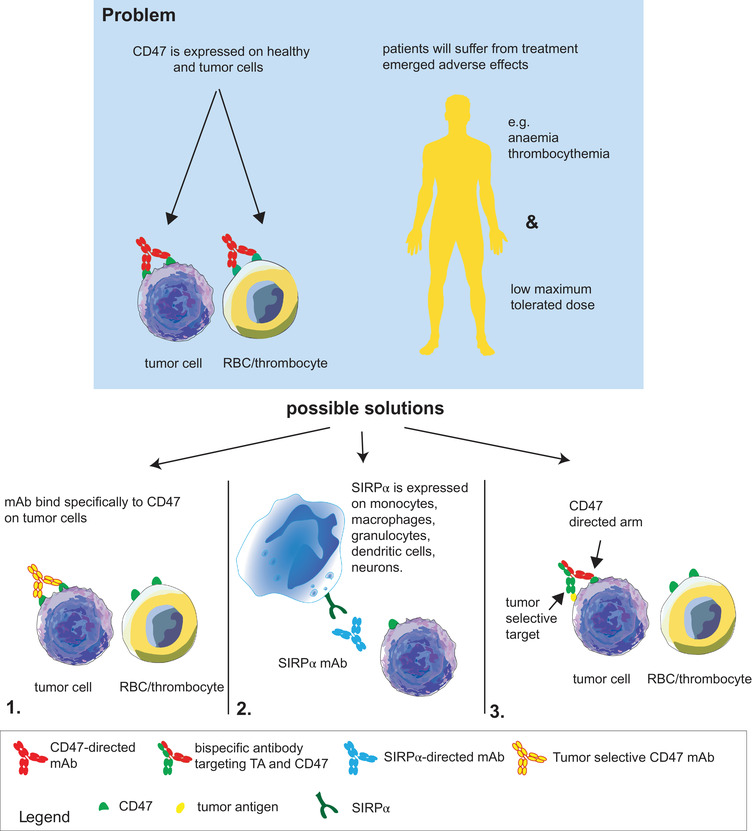
Toxicity observed with CD47‐signal regulatory protein alpha (SIRPα) blocking antibodies and strategies to improve them. CD47 is expressed on healthy and tumour cells, and therefore, targeting CD47 will also result in the loss of healthy cells with CD47 expression, such as red blood cells (RBCs) and thrombocytes. The thrombocythemia and anaemia that are the result of these ‘off‐target’ effects result in a low maximum tolerated dose in clinical trials, thus limiting the effects on the tumour. To overcome this, three different strategies are discussed in this review. (1) Novel body formats are designed to target only CD47 expressed on cancer cells. These antibodies are designed to bind to clustered CD47 only. Another method to prevent binding to RBCs is by designing an antibody that binds to the epitope of CD47 that is closely located to an N‐glycosylated on RBCs and therefore functions as a ‘shield’ for RBCs. (2) Instead of targeting CD47 with CD47 monoclonal antibody (mAb) or recombinant human SIRPα (rhSIRPα), it is also possible to target SIRPα on phagocytes, thereby circumventing the RBCs and thrombocytes. (3) Bispecific antibodies are designed to target CD47 only to tumour cells with a second arm that binds only to tumour‐selective targets

Many cancers misuse this signalling axis to escape immunosurveillance by phagocytes.^3^ Correspondingly, high expression of CD47 is associated with poor prognosis in a range of cancers.^4^, ^5^, ^6^ To overcome escape of immunosurveillance via CD47‐SIRPα signalling, a series of immunotherapeutics is being evaluated clinically (Table 1). Fundamentally, they can be separated into strategies that target and functionally block CD47, using CD47 antibodies or recombinant forms of human SIRPα, and strategies that target and block SIRPα with SIRPα antibodies or soluble CD47 (Figure 1, panel 1). These approaches are analogous but do differ in selectivity as CD47 is expressed on most if not all cancerous and normal tissues. In contrast, the expression of SIRPα is limited to specific innate cell types, most notably macrophages, monocytes, granulocytes, dendritic cells, neutrophils and neurons^7^ (Figure 1, panel 2). Immunotherapeutic approaches that block the CD47‐SIRPα signalling axis have yielded promising preclinical activity, with prominent induction of phagocytosis in various cancer types.^8^, ^9^, ^10^ In a preclinical setting, CD47‐SIRPα blocking is sufficient to trigger phagocytosis as a single agent.^1^, ^11^ However, single‐agent treatment with CD47‐SIRPα blocking therapeutics in patients is associated with a lack of therapeutic effect at the maximum tolerated dose (MTD) and significant dose‐limiting toxicity (DLT), most notably anaemia and thrombocytopenia.^12^, ^13^, ^14^

**TABLE 1:** Overview of all CD47‐signal regulatory protein alpha (SIRPα) blocking antibodies that are or have been in clinical trials

Name	Format	Target	Isotype	Cotreatment	Type of cancer	Progress	Start date	(Expected) completion date	NCT/CRT	Company
AK117^26^	CD47 mAb	CD47	IgG4	Azacitidine	Acute myeloid leukaemia	Phase I/II clinical	Jun‐21		CTR20211305	Akeso
AK117^26^	CD47 mAb	CD47	IgG4	Monotherapy	Advanced cancer	Phase I clinical	Jan‐21	1‐Jan‐23	NCT04728334	Akeso
AK117^26^	CD47 mAb	CD47	IgG4	Monotherapy	Advanced solid cancer, non‐Hodgkin lymphoma	Phase I clinical	Dec‐20		CTR20202684	Akeso
AK117^26^	CD47 mAb	CD47	IgG4	Azacitidine	Myelodysplastic syndrome	Phase I/II clinical	May‐21	May‐24	NCT04900350	Akeso
AK117^26^	CD47 mAb	CD47	IgG4	Azacitidine	Myelodysplastic syndrome	Phase I/II clinical	May‐21		CTR20210825	Akeso
AO‐176^25^	CD47 mAb	CD47	IgG2	Monotherapy, paclitaxel, pembrolizumab	Advanced solid cancer	Phase I/II clinical	Feb‐19	Mar‐23	NCT03834948	Arch Oncology
AO‐176^25^	CD47 mAb	CD47	IgG2	Monotherapy, bortezomib, bortezomib + dexamethasone	Multiple myeloma	Phase I/II clinical	Nov‐20	Mar‐23	NCT04445701	Arch Oncology
CC‐90002^14^	CD47 mAb	CD47	IgG4	Monotherapy	Acute myeloid leukemia, myelodysplastic syndrome	Phase I clinical	Mar‐16	Ended preliminary	NCT02641002	Celgene
CC‐90002^14^	CD47 mAb	CD47	IgG4	Rituximab	Haematological cancer	Phase I clinical	Mar‐15	Feb‐22	NCT02367196	Celgene
CPO107/JMT601	CD20/CD47	CD47	bsAb	Monotherapy	Advanced CD20‐positive non‐Hodgkin lymphoma	Phase I clinical	Aug‐21	Aug‐24	NCT04853329	Conjupro Biotherapeutics
DSP‐107^110^	rhSIRPα/4‐1BB	CD47	bsAb	Monotherapy, atezolizumab	Advanced solid cancer, non‐small cell lung cancer	Phase I clinical	Oct‐20	Aug‐23	NCT04440735	Kahr medical
Hu5F9‐G4 (Magrolimab)^18^	CD47 mAb	CD47	IgG4	Cetuximab	Advanced solid cancer and advanced colorectal cancer	Phase I/II clinical	Nov‐16	10‐Feb‐20	NCT02953782	Stanford University Forty Seven
Hu5F9‐G4 (Magrolimab)^18^	CD47 mAb	CD47	IgG4	Rituximab	B‐cell non‐Hodgkin lymphoma	Phase I/II clinical	Nov‐16	Nov‐21	NCT02953509	Stanford University Forty Seven
Hu5F9‐G4 (Magrolimab)^18^	CD47 mAb	CD47	IgG4	Obinutuzumab, venetoclax	Follicular lymphoma, marginal zone lymphoma, mantle cell lymphoma	Phase I clinical	Oct‐20	Jun‐21	NCT04599634	National cancer institute (NCI)
Hu5F9‐G4 (Magrolimab)^18^	CD47 mAb	CD47	IgG4	Azacitidine	Haematological cancer	Phase I clinical	Sep‐17	Aug‐21	NCT03248479	Stanford University Forty Seven
Hu5F9‐G4 (Magrolimab)^18^	CD47 mAb	CD47	IgG4	Pembrolizumab	Hodgkin lymphoma	Phase II clinical	Nov‐21	Dec‐22	NCT04788043	Stanford University Forty Seven
Hu5F9‐G4 (Magrolimab)^18^	CD47 mAb	CD47	IgG4	Avelumab	Ovarian cancer	Phase I clinical	May‐18	1‐Dec‐20	NCT03558139	Stanford University Forty Seven
HX009^105^	CD47‐PDL‐1	CD47	bsAb	Monotherapy	Advanced solid cancer	Phase II clinical	Jun‐21		CTR20211292	Waterstone Hanxbio Pty Ltd.
HX009^105^	CD47‐PDL‐1	CD47	bsAb	Monotherapy	Advanced solid cancer	Phase I clinical	Jun‐19	Sep‐21	NCT04097769	Waterstone Hanxbio Pty Ltd.
IBI188 (Letaplimab)	CD47 mAb	CD47	IgG4	Azacitidine, decitabine	Acute myeloid leukaemia	Phase 1/2 clinical	Jul‐20		CTR20200938	Innovent Biologics
IBI188 (Letaplimab)	CD47 mAb	CD47	IgG4	Azacitidine	Acute myeloid leukaemia and myelodysplastic syndromes	Phase I clinical	Apr‐21		CTR20210761	Innovent Biologics
IBI188 (Letaplimab)	CD47 mAb	CD47	IgG4	Monotherapy, rituximab	Advanced cancer	Phase I clinical	Dec‐18	Jan‐22	NCT03717103	Innovent Biologics
IBI188 (Letaplimab)	CD47 mAb	CD47	IgG4	Monotherapy	Advanced solid cancer	Phase I clinical	Nov‐18		CTR20182140	Innovent Biologics
IBI188 (Letaplimab)	CD47 mAb	CD47	IgG4	Monotherapy	Advanced solid cancer and lymphoma	Phase I clinical	Jan‐19	Aug‐22	NCT03763149	Innovent Biologics
IBI188 (Letaplimab)	CD47 mAb	CD47	IgG4	Azacitidine	Myelodysplastic syndrome	Phase 1/3 clinical	Jul‐20		CTR20201039	Innovent Biologics
IBI‐322^104^	CD47‐PD‐1	CD47	IgG	PD‐1/CD47 bsAb, single agents	Advanced solid cancer	Phase I clinical	Jun‐21	Jun‐23	NCT04912466	Innovent Biologics
IBI‐322^104^	CD47‐PD‐1	CD47	IgG	PD‐1/CD47 bsAb, single agents	Advanced solid cancer	Phase I clinical	Apr‐21	Dec‐22	NCT04338659	Innovent Biologics
IBI‐322^104^	CD47‐PD‐1	CD47	IgG	PD‐1/CD47 bsAb, single agents	Advanced solid cancer	Phase I clinical	Jul‐20	Sep‐23	NCT04328831	Innovent Biologics
IBI‐322^104^	CD47‐PD‐1	CD47	IgG	PD‐1/CD47 bsAb, single agents	Haematological cancer	Phase I clinical	May‐21	Nov‐23	NCT04795128	Innovent Biologics
IBI‐322^104^	CD47‐PD‐1	CD47	IgG	PD‐1/CD47 bsAb, azacitidine/decitabine	Myeloid tumour	Phase I clinical	Dec‐21	Jun‐23	NCT05148442	Innovent Biologics
IMC‐002^8^	CD47 mAb	CD47	IgG4	Monotherapy	Metastatic/locally advanced solid tumours, relapsed or refractory lymph	Phase I clinical	Jun‐20	Dec‐22	NCT04306224	ImmuneOncia
IMM0306	CD47‐CD20	CD47	IgG1	Monotherapy	Non‐Hodgkin lymphoma	Phase I clinical	May‐20		CTR20192612	ImmuneOnco Biopharma
IMM2902	HER2/rhSIRPα	CD47	bsAb	Monotherapy	Advanced solid cancer	Phase I clinical	Jan‐22	Jun‐23	NCT05076591	ImmuneOnco Biopharma
PF‐07257876	CD47/PD‐L1	CD47	bsAb	Monotherapy	Non‐small cell lung cancer, squamous cell carcinoma of the head and neck	Phase I clinical	Jun‐21	Apr‐24	NCT04881045	Pfizer
SGN‐CD47 M	ADC‐CD47	CD47	Unknown	Monotherapy	Advanced solid cancer	Phase I clinical	Jul‐19	Sep‐20	NCT03957096	Seagen
SHR‐1603	CD47 mAb	CD47	IgG4		Advanced solid cancer	Phase I clinical	Nov‐18	On hold	CTR20181964	Jiangsu HengRui Medicine
SRF231^13^	CD47 mAb	CD47	IgG4	Monotherapy	Advanced cancer	Phase I/Ib clinical	Apr‐18	1‐9‐2020 ended without results	NCT03512340	Surface Oncology
STI‐6643	CD47 mAb	CD47	IgG4	Monotherapy	Advanced solid cancer	Phase I clinical	Aug‐21	Dec‐22	NCT04900519	Sorrento therapeutics
TBQ2928	Unknown	CD47	Unknown	Monotherapy	Advanced cancer	Phase I clinical	Aug‐21	Dec‐22	NCT04854681	Chia Tai Tianqing Pharmaceutical Group
TG‐1801^43^	CD47‐CD19	CD47	IgG1 bsAb	Ublituximab, umbralisib	B‐cell lymphoma	Phase I clinical	Mar‐19	1‐Aug‐21	NCT03804996	Novimmune TG Therapeutics
TG‐1801^43^	CD47‐CD19	CD47	IgG1 bsAb	Ublituximab	B‐cell lymphoma, chronic lymphocytic leukaemia	Phase I clinical	Apr‐21	Dec‐23	NCT04806035	Novimmune TG Therapeutics
TI‐061^15^	CD47 mAb	CD47	IgG4	Pembrolizumab	Solid cancer and breast cancer	Phase I/II clinical		Ended preliminary		Arch Oncology
TJ011133 (Lemzoparlimab)^27^	CD47 mAb	CD47	IgG4	Monotherapy	Acute myeloid leukaemia and myelodysplastic syndromes	Phase I/II clinical	Mar‐20	Aug‐23	NCT04202003	I‐MAB Biopharma
TJ011133 (Lemzoparlimab)^27^	CD47 mAb	CD47	IgG4	Monotherapy	Acute myeloid leukaemia and myelodysplastic syndrome	Phase I/II clinical	Dec‐12		CTR20192522	I‐MAB Biopharma
TJ011133 (Lemzoparlimab)^27^	CD47 mAb	CD47	IgG4	Azacitidine	Acute myeloid leukaemia/myelodysplastic syndrome	Phase I/II clinical	Mar‐21		CTR20210555	I‐MAB Biopharma
TJ011133 (Lemzoparlimab)^27^	CD47 mAb	CD47	IgG4	Pembrolizumab, rituximab	Advanced solid cancer and lymphoma	Phase I clinical	May‐19	Sep‐23	NCT03934814	I‐MAB Biopharma
TJ011133 (Lemzoparlimab)^27^	CD47 mAb	CD47	IgG4	Rituximab	Lymphoma, CD20+	Phase I clinical	Mar‐21		CTR20210313	I‐MAB Biopharma
TTI‐621^12^	SIRPα‐Fc	CD47	IgG1	PD‐1/PD‐L1 inhibitor, pegylated interferon α2a, ta	Advanced solid cancer and mycosis fungoides	Phase I clinical	Sep‐16	Mar‐20	NCT02890368	Trillum Therapeutics
TTI‐621^12^	SIRPα‐Fc	CD47	IgG1	Monotherapy, rituximab, nivolumab	Haematological cancer and selected solid tumours	Phase I/Ib clinical	Jan‐16	Dec‐21	NCT02663518	Trillum Therapeutics
TTI‐621^12^	SIRPα‐Fc	CD47	IgG1	Doxorubicin	Leiomyosarcoma	Phase I/II clinical	Jun‐21	Jun‐23	NCT04996004	Trillum Therapeutics
TTI‐622^19^	SIRPα‐Fc	CD47	IgG4	Rituximab, PD‐1 inhibitor, proteasome inhibitor re	Lymphoma or myeloma	Phase I clinical	May‐18	30‐Apr‐22	NCT03530683	Trillum Therapeutics
ZL‐1201	CD47 mAb	CD47	IgG4	Monotherapy	Advanced cancer	Phase I clinical	May‐21		CTR20210973	Zai Lab
ZL‐1201	CD47 mAb	CD47	IgG4	Monotherapy	Advanced cancer	Phase I clinical	May‐20	1‐Jan‐24	NCT04257617	Zai Lab
ALX148 (Evorpacept)^32^	SIRPα‐D1	SIRPα	IgG1 (inactive)	Venetoclax, azacitidine	Acute myeloid leukemia	Phase I/II clinical	May‐21	Dec‐23	NCT04755244	ALX oncology
ALX148 (Evorpacept)^32^	SIRPα‐D1	SIRPα	IgG1 (inactive)	Pembrolizumab, trastuzumab, rituximab, ramucirumab	Advanced solid cancer and lymphoma	Phase I clinical	Feb‐17	Dec‐21	NCT03013218	ALX oncology
ALX148 (Evorpacept)^32^	SIRPα‐D1	SIRPα	IgG1 (inactive)	Trastuzumab, ramucirumab, paclitaxel	Gastric cancer, gastroesophageal junction adenocarcinoma, gastric adenocarcinoma	Phase II/III clinical	Aug‐21	Jul‐26	NCT05002127	ALX oncology
ALX148 (Evorpacept)^32^	SIRPα‐D1	SIRPα	IgG1 (inactive)	Pembrolizumab, 5‐fluoruracil, cisplatin	Head and neck cancer	Phase 2 clinical	Jun‐42	Oct‐24	NCT04675333	ALX oncology
ALX148 (Evorpacept)^32^	SIRPα‐D1	SIRPα	IgG1 (inactive)	Rituximab, lenalidomide	Indolent and aggressive B‐cell non‐Hodgkin lymphoma	Phase I/II clinical	Sep‐21	Mar‐26	NCT05025800	M.D. Anderson Cancer Center
ALX148 (Evorpacept)^32^	SIRPα‐D1	SIRPα	IgG1 (inactive)	Azacitidine	Myelodysplastic syndrome	Phase I/II clinical	Oct‐20	Dec‐23	NCT04417517	ALX oncology
BI765063/OSE‐172^36^	SIRPα‐D1	SIRPα	IgG1	PD‐1 antagonist	Advanced solid cancer	Phase I clinical	Apr‐19	Dec‐22	NCT03990233	OSE Immunotherapeutics
CC‐95251	SIRPα	SIRPα		Rituximab, cetuximab	Advanced cancer	Phase I	Feb‐19	Nov‐24	NCT03783403	Celgene
IMM01	SIRPα	SIRPα	IgG1	Monotherapy	Non‐Hodgkin lymphoma	phase I clinical	Aug‐21	Jan‐22	CTR20191531	ImmuneOnco Biopharma
SL‐172154^108^	SIRPα‐CD40	SIRPα	Fusion protein	Monotherapy	Gynaecological tumours	Phase I clinical	Jun‐20	Jun‐22	NCT04406623	Shattuck lab
SL‐172154^108^	SIRPα‐CD40	SIRPα	Fusion protein	Monotherapy	Skin, head and neck cancer	Phase I clinical	Sep‐20	Jul‐22	NCT04502888	Shattuck lab

*Note*: These antibodies are designed with different Fc domains as described in the fourth column. Within these clinical trials, CD47/SIRPα blockade is often evaluated with cotreatment with other monoclonal antibodies, chemotherapy, demethylating agents and/or proteosome inhibitors. Clinical trials with CD47 blocking are conducted in a host of different tumour types.

Abbreviations: bsAb, bispecific antibody; HER2, human epidermal growth factor receptor 2; Ig, immunoglobulin; mAb, monoclonal antibody; PD‐(L)1, programmed cell death (ligand)1; (rh)SIRPα, (recombinant human)SIRPα.

*Source*: CD47‐related clinical trials registered in US national clinical trials registry (NCT) system (www.clinicaltrials.gov) or in the China drug trials registry (CDT) system (www.chinadrugtrials.org.cn).

**FIGURE 2:**
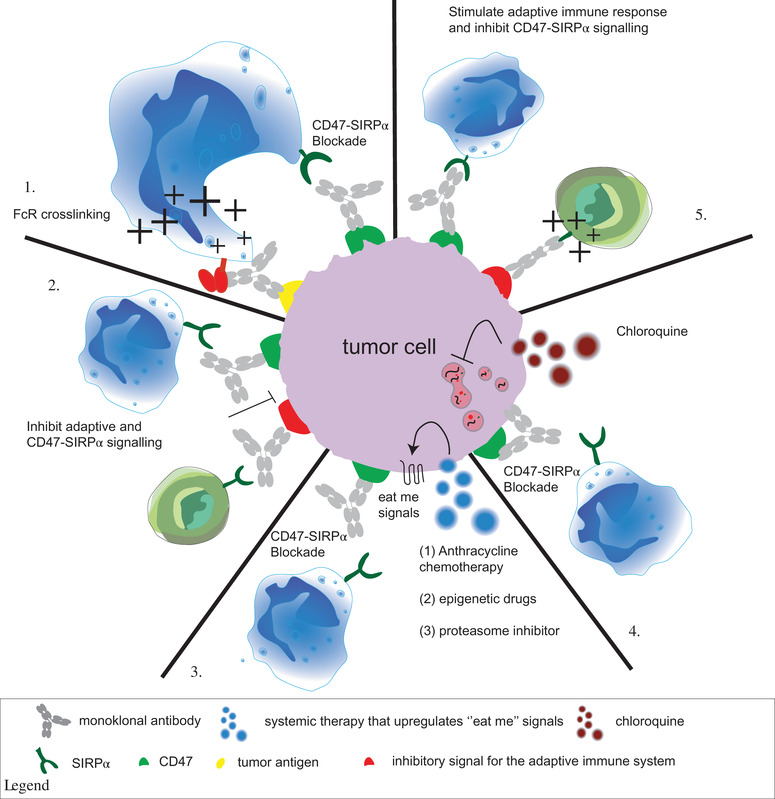
Combinatory strategies that improve the therapeutic effect of CD47‐signal regulatory protein alpha (SIRPα) blocking. Currently, five different strategies to improve CD47 blocking therapy are being evaluated. (1) In clinical trials with several different types of cancer parents, Fc receptor (FcR) crosslinking antibodies that directly target tumour antigens are combined with CD47 blocking. The combination of CD47 blocking with (5) stimulation of adaptive costimulatory signals or (2) blockade of adaptive ‘don't eat me’ signals is also a promising combinatory strategy, as increasing evidence states that the adaptive immune system has a pivotal role in the effect of CD47 blocking therapy. (3) The combination of anthracycline, epigenetic drugs (demethylating agents) and proteasome inhibitors also improved the therapeutic effect of CD47 blockade. Most likely, this improvement is at least partly caused by upregulation of ‘eat me’ signals on tumour cells triggering phagocytosis by immune cells. Finally, accumulating evidence points to CD47‐SIRPα blocking antibodies triggering not only phagocytosis but also autophagy of tumour cells and health issues. (4). Combination with autophagy blockers seems to improve the phagocytic index in non‐small cell lung cancer (NSCLC) and glioblastoma in a preclinical setting

In this review, we first provide an overview of the clinical results of CD47‐SIRPα therapeutics with a focus on the limitations encountered, most notably toxicity (for an overview, see Figure 1). We then review the development of various novel monoclonal and bispecific antibody (bsAb) formats designed to overcome these limitations and improve the efficacy of CD7‐SIRPα targeting. Subsequently, we detail (pre)clinical studies in which CD47 targeting is rationally combined with other therapeutics, such as opsonizing antibodies, hypomethylating agents or proteasome inhibitors, in order to shift the phagocytic balance towards cancer cell removal and achieve synergistic anticancer activity (Figure 2). Finally, we discuss efforts to combine activation of immunity upon CD47 blockade with (re)activation of anticancer T‐cell immunity using bsAbs.

## 2 LIMITATIONS OF CD47‐SIRPα‐TARGETING THERAPEUTICS ENCOUNTERED IN CLINICAL SETTINGS

In clinical trials with CD47 therapeutics, a diverse spectrum and severity of toxicities have been encountered depending on the antibody used. Specifically, the first clinical trial with CD47 antibody TI‐061, having an IgG4 isotype, was halted after the first inclusion resulted in a fatality.^15^ In subsequent clinical trials with magrolimab (also an IgG4‐based CD47 antibody), the major yet manageable side effects in various cancer types were anaemia and infusion‐related reactions that did not lead to an MTD.^16^, ^17^, ^18^ Importantly, the occurrence of anaemia upon magrolimab treatment was significantly reduced by implementation of a so‐called priming dose of 1 mg/kg followed by a maintenance dose of 10–30 mg/kg.^17,18^ However, the ENHANCE‐2 trial in which magrolimab is combined with venetoclax and azacytidine to treat acute myeloid leukaemia (AML) and myelodysplastic syndrome (MDS) recently halted patient inclusion due to unexpected serious adverse reactions. Moreover, a clinical trial with the IgG4‐based CD47 antibody CC‐90002 in relapsed/refractory AML or high‐risk MDS (23/28) was discontinued after 82% of patients developed febrile neutropenia and four patients developed grade 4 toxicity.^14^ In this study, treatment with CC‐90002 did not yield objective responses. CC‐90002 was also evaluated in non‐Hodgkin lymphoma (NHL) patients in combination with RTX, which was again associated with significant toxicity, namely, grade 3 or 4 anaemia, neutropenia and thrombocytopenia.^4^ The overall response rate (ORR) was (5/37) 13%, and stable disease (SD) was observed in (9/37) 25% of patients.^4^ Similarly, the IgG4‐based antibody SRF231 triggered grade 4 thrombocytopenia, grade 4 amylase and lipase increase and grade 3 fatigue in advanced malignancies.^13^ These DLTs resulted in an MTD of 4 mg/kg weekly, with no objective responses.

This difference in the toxicity profile of antibodies even with the same isotype clearly suggests that antibody affinity and/or respective epitope on CD47 may partly dictate the toxicity of mAb treatment. Nevertheless, the isotype can also contribute to the toxicity and efficacy of CD47 blocking. For instance, the IgG1 containing SIRPαFc fusion protein TTI‐621 triggered grade 4 transient thrombocytopenia and had a relatively low (0.2 mg/kg) MTD.^12^ In contrast, the analogous IgG4 containing protein TTI‐622 triggered limited mostly grade 1 or 2 adverse events (AEs) at doses up to 8 mg/kg.^19^ At the MTD, TTI‐621 only triggered a 13% ORR upon single treatment of diffuse large B‐cell lymphoma (DLBCL) patients,^12^ with TTI‐622 having a 20% objective response rate.^19^ This difference in toxicity of TTI‐621 and TTI‐622 is in line with expectations that the IgG1 Fc tail triggers the FcR‐mediated immune response more than an IgG4 Fc domain.^20^

From the above, it is clear that although CD47 targeting can effectively (re)activate anticancer immunity off‐tumour, yet on‐target activity/toxicity is a major concern for CD47‐SIRPα blocking that limits therapeutic applicability. Therefore, the design of novel antibody formats that do not bind or have a reduced binding profile to RBCs or that have increased selectivity for cancer cells is of major interest.

## 3 STRATEGIES TO REDUCE THE INTERACTION OF CD47/SIRPα BLOCKERS WITH RBCS

An important means to improve the therapeutic index of CD47‐SIRPα blocking is to reduce binding or prevent depletion of young RBCs. Simultaneously, strong binding to CD47 on cancer cells must be retained. Such an improved binding profile may be achievable for various reasons. Firstly, on the membrane of young RBCs, CD47 is localised in a distinct complex with RhAG, protein 4.2, Band 3 and the cytoskeleton, which prevents CD47 from clustering.^21^ When RBCs age, CD47 can bind to thrombospondin (TSP) or TSP‐like peptide (4N1K) and thereby colocalise with gangliosides (a component of lipid rafts) and clusters. On the surface of tumour cells, CD47 binds TSP during the whole lifespan,^2^, ^22^ creating a distinct binding profile that may enable ‘untargeting’ of young RBCs. Furthermore, CD47 can be heavily glycosylated in a cell‐type specific manner, with five potential NXT/S sequences in its extracellular IgV domain that are potentially modified by glycosaminoglycans.^23^ As glycosylation patterns are often uniquely altered in cancer,^24^ cancer‐specific glycosylation patterns may be used to selectively target cancer‐expressed CD47.

Prominent examples that such altered binding characteristics can be achieved are next‐generation antibodies, such as Lemzoparlimab, AK117, IMC‐002, AO‐176 and STI‐6643 that have reduced binding to RBCs, B cells, T cells and natural killer (NK) cells.^8^, ^25^, ^26^ Lemzoparlimab binds to a distinct conformational epitope closely located to an N‐linked CD47 glycosylation site. On RBCs, this N‐linked glycan structure is hypothesised to function as a ‘shield’ and prevent lemzoparlimab binding to human RBCs. In line with this, deglycosylation by peptide‐N‐glycosidase (PNGase) treatment restored RBC binding.^27^ Of these next‐generation antibodies, clinical results have only been reported for lemzoparlimab and AK117, with no serious haematological adverse effects or DLTs observed in lemzoparlimab doses up to 20–30 mg/kg weekly.^28^ Treatment with AK117 similarly did not associate with haematological adverse effects, even up to 20 mg/kg.^26^ Moreover, among seven evaluable relapsed/refractory NHL patients, three had a complete response (CR), one a partial response and three had SD when treated with lemzoparlimab in combination with rituximab.^29^

In contrast to CD47 antibodies, the recombinant human (rh)SIRPα‐based therapeutic TTI‐621 did not trigger anaemia in clinical studies.^12^ A hypothesis for this observation is that rhSIRPα only efficiently binds to clustered CD47, whereas RBC‐expressed CD47 does not form clusters.^30^ In support of this hypothesis, clustering of CD47 on RBCs using a non‐competing CD47 antibody restored the binding of TTI‐621 to RBCs.^31^ In clinical trials, treatment with TTI‐621 and ALX148 (SIRPα fusion protein) resulted in thrombocytopenia but not anaemia.^12^, ^32^ Thrombocytopenia was postulated as an on‐target effect of CD47 blocking together with opsonisation by activating IgG1, resulting in platelet removal by macrophages.^12^

An alternate approach to circumvent RBC toxicity is the targeting of the ligand SIRPα, of which expression is restricted to cells of the myeloid lineage. Herewith, the CD47 ‘antigen sink’ encountered with CD47‐targeting agents is also circumvented,^33^ as validated in vitro.^34^ Most SIRPα mAbs induce antibody‐dependent cellular phagocytosis (ADCP) of cancer cells in combination with opsonizing antibodies that target tumour antigens. Moreover, novel anti‐SIRPα Abs have been developed to block CD47/SIRPα signalling and simultaneously induce internalisation of the SIRPα/mAb complex, leading to downregulation of phagocyte‐expressed SIRPα or prevention of SIRPα clustering.^35^ However, whether the blockade of SIRPα also impacts on the phagocytosis of other healthy cells, for example, RBCs, requires further investigation. Several anti‐SIRPα antibodies are in preclinical development, while CC‐95251 (NCT03783403) and BI 765063 (NCT03990233) are in early clinical trials. In the first clinical trial with BI 765063, treatment only induced mild infusion‐related AEs and no anaemia or thrombocytopenia. Clinical benefit was observed in 21/47 (45%) patients with advanced solid tumours.^36^ Thus, circumvention of anaemia or thrombocytopenia can be accomplished with various strategies, including the development of CD47 mAbs that bind to a distinct epitope that is not targetable on RBCs or by targeting SIRPα.

### 3.1 Target antigen‐directed blocking of CD47

**FIGURE 3:**
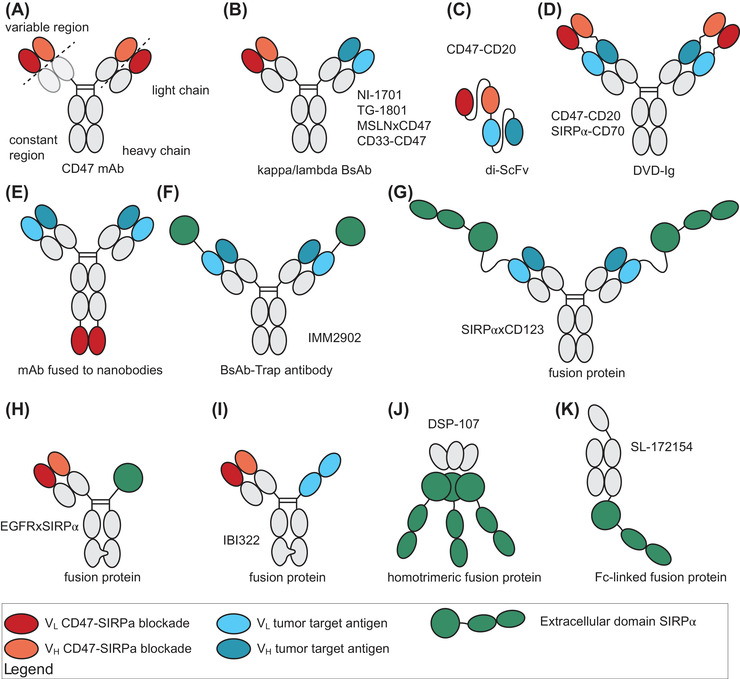
Different bifunctional CD47‐targeting antibodies (bispecific antibodies, bsAbs) and fusion proteins compromising signal regulatory protein alpha (SIRPα). (A) A normal monoclonal antibody (mAb) demonstrating the different regions used in bifunctional proteins. (B) Kappa/lambda bsAb compromised of a kappa and lambda light chain and are fused by a common heavy chain with an active IgG1 domain. (C) Di‐single‐chain variable fragment (di‐ScFv) is compromised of the variable regions of RTX and CD47 mAb fused with a linker. (D) The dual‐variable‐fragment domain is compromised of a full antibody fused to the variable region of a CD47‐ or SIRPα‐directed antibody. (E) A full RTX antibody is fused to a CD47‐directed nanobody (a single‐domain antibody fragment derived from a naturally occurring heavy‐chain IgG antibody). (F) A bispecific trap antibody that is comprised of a human epithelial growth factor receptor 2 (HER2)‐directed full antibody and the variable domain of the extracellular domain (ED) of SIRPα. (G) A fusion protein comprising the full CD123 antibody fused to the ED of SIRPα. (H) A fusion protein compromised of the V_H_ V_L_ of an epidermal growth factor receptor (EGFR) antibody fused to the ED of SIRPα using the knobs‐into‐holes technique. (I) A fusion protein compromised of V_H_ V_L_ of a CD47 mAb fused with a programmed cell death ligand 1 (PD‐L1) mAb that consists of an Fc domain with two V_L_. (J) A homotrimeric fusion protein compromised of the ED of 4‐1BBL and three EDs of SIRPα. (K) An Fc‐linked fusion protein compromised of the ED of SIRPα that is linked through an inactive IgG4 Fc domain with the ligand of CD40. DVD‐Ig, dual‐variable domain immunoglobulin; MSLN, mesothelin; V_H_, variable domain of the heavy chain; V_L_, variable domain of the light chain

An alternate solution for reducing toxicity and possibly increasing efficacy is the development of bispecific CD47‐targeting antibodies (bsAbs) or bifunctional fusion proteins comprising SIRPα. In bsAbs, one arm of the antibody recognises CD47, whereas the other arm recognises a target antigen that can direct the antibody to the cell of interest. An important feature of bsAbs is higher avidity to dual antigen‐expressing cells relative to single antigen‐expressing cells.^37^ Depending on the format, a bsAb will have different pharmacokinetics and effector functions. For example, a human IgG1 Fc domain can activate myeloid immune effector cells via Fcγ receptors, whereas an IgG4 Fc domain cannot.^38^ Various bispecific approaches to block CD47‐SIRPα signalling specifically on selected cancer cell types have been developed (see Table 2 for overview and Figure 3 for formats being used). Currently, three such bispecific antibodies that target CD47 to the B‐cell antigen CD19 or CD20 are being evaluated in clinical trials (NCT03804996, NCT04853329 and NCT04853329). Furthermore, bifunctional immunomodulatory approaches have been developed to combine CD47 blocking with additional immune‐activating therapeutics. In the next sections, the rationale and development are discussed.

**TABLE 2:** Overview of bispecific approaches that block CD47‐signal regulatory protein alpha (SIRPα) signalling

Name	Target	CD47/SIRPα blocking arm	Format	Cotreatment	Type of cancer	Progress
NI‐1701	CD19	CD47	κλ‐bispecific format^42^	Rituximab	Lymphoma	Preclinical
TG‐1801	CD19	CD47	κλ‐bispecific format^43^	Umbralisib, ublituximab	Lymphoma	Clinical
	CD20	CD47	scFv fusion protein^46^	Obinutuzumab, daratumumab, alemtuzumab	Lymphoma	Preclinical
	CD20	CD47	CD47 nanobody fused to C‐terminal of rituximab^48^		Lymphoma	Preclinical
CPO107/JMT601	CD20	rhSIRPα	IgG 1 Fc domain		CD20+ lymphoma	Clinical
HMBD004	CD33	CD47	1 + 1 IgG format^49^		Acute myeloid leukaemia	Preclinical
	CD33	rhSIRPα	N‐terminal SIRPα fused to the variable light chain CD33‐targeting IgG1^52^		Acute myeloid leukaemia	Preclinical
	CD123	rhSIRPα	The extracellular domain SIRPα was fused to CD123 antibody^54^		Acute myeloid leukaemia	preclinical
NI‐1801	MSLN	CD47	CD47xMSLN with functional IgG1 Fc^55^		Hepatocellular carcinoma	Preclinical
	MSLN	CD47	High‐affinity MSLN Ab^57^		Hepatocellular carcinoma, ovarian carcinoma, gastric carcinoma	Preclinical
	CD70	rhSIRPα	Variable domain CD70 Ab with variable domain SIRPα^33^		Renal cell carcinoma, Burkitt lymphoma	Preclinical
	EGFR	rhSIRPα	Knobs‐into‐holes: EGFR mAb was fused to a SIRPα variant with high affinity to CD47^61^		Squamous cell carcinoma	Preclinical
IMM2902	HER2	rhSIRPα	Trap antibody receptor fusion protein		Advanced solid cancer	Clinical
PF‐07257876	PD‐L1	CD47	Unknown		Non‐small cell lung cancer, squamous cell carcinoma of the head and neck, ovarian c	Clinical
IMM0306^47^	CD20	CD47	DVD‐Ig^47^	Rituximab	Non‐Hodgkin lymphoma	Clinical
IBI‐322	PD‐1	CD47	IgG4 Fc domain^104^	Azacitidine/decitabine (with myeloid malignancies)	Advanced solid cancer, haematological cancer, myeloid tumours	Clinical
HX009	PD‐L1	CD47	IgG4‐Fc region of anti‐PD‐1 mAb and the extracellular domain of SIRPα^105^		Advanced solid cancer	Clinical
DSP‐107	4‐1BB	CD47	Fusion of the extracellular domain of SIRPα genetically fused to the extracellular domain of 4‐1BBL^110^	Atezolizumab	Advanced solid cancer, non‐small cell lung cancer	Clinical
SL‐172154	CD40	rhSIRPα	Fusion protein consist of human SIRPα and CD40 L linked via a human Fc^108^		Gynaecological tumours, skin, head and neck cancer	Clinical

*Note*: These bispecific approaches are either in the preclinical or clinical phase of development. Interestingly, they are designed in various formats and target a variety of tumour antigens.

Abbreviations: DVD‐Ig, dual‐variable domain immunoglobulin; EGFR, endothelial growth factor receptor; HER2, human epithelial growth factor receptor 2; Ig, immunoglobulin; mAb, monoclonal antibody; MSLN, mesothelin; PD‐(L)1, programmed death receptor (ligand) 1; (rh)SIRPα, (recombinant human)SIRPα; ScFv, single‐chain variable fragment.

### 3.2 B‐cell malignancies

For the selective targeting of CD47 blocking to malignant B cells, both CD19 and CD20 have been exploited as target antigens. CD19 is expressed on almost all B cells and has proven to be a good target for the treatment of relapsed/refractory DLBCL patients with chimeric antigen receptor (CAR) T‐cell therapy.^39^ CD20 is a B‐cell lineage‐specific antigen expressed on the cell surface of most B‐cell lymphomas.^40^ CD20‐targeting antibodies such as RTX have been broadly integrated into the standard‐of‐care for B‐cell lymphoma.^41^ The bsAb NI‐1701 was designed for the tumour‐selective targeting of CD47 to CD19. The κλ‐body format comprises a humanised IgG1 Fc heavy chain, with a κ light chain recognising CD47 and a λ light chain recognising CD19.^42^ Notably, the affinity of NI‐1701 towards CD19 is higher than that for CD47 (500 nM vs. 0.6 nM). Correspondingly, NI‐1701 only detectably bound to B cells and not to RBCs in whole blood in vitro. NI‐1701 inhibited the growth of Raji tumours in NOD/SCID mice significantly better than CD47 or RTX single treatment. Moreover, NI‐1701 treatment potentiated the effect of RTX treatment, with 92% inhibition of tumour growth compared to 72% and 48% for NI‐1701 and RTX single treatment, respectively.^42^ Another CD47xCD19 κλ bsAb (TG‐1801) is humanised IgG1 that minimally increases ADCP upon monotherapy in vitro. Importantly, the CD47 arm, which has a similar affinity to human and cynomolgus CD47, did not induce any haematological AEs in cynomolgus monkeys.^43^ TG‐1801 was also evaluated in combination with umbralisib, a phosphatidylinositol‐3‐kinase and CK1ε inhibitor, and ublituximab, a glycoengineered CD20 mAb.^44^, ^45^ Interestingly, cotreatment with TG‐1801, ublituximab and umbralisib inhibited tumour growth by 93% in a Burkitt lymphoma mouse model compared to 76% as a single treatment. Currently, TG‐1801 is being evaluated in a clinical trial (NCT03804996) with this combination.

CD20‐selective inhibition of the CD47‐SIRPα interaction was evaluated with several different antibody formats. For example, we generated a bsAb fragment comprised of the single‐chain variable fragment (scFv) of a CD47 mAb fused to the scFv of RTX. This bi‐scFv only blocked SIRPα binding to CD47 on CD47+/CD20+ double‐positive cell lines and not on CD47 single‐positive cell lines. Correspondingly, phagocytosis upon single agent treatment as well as upon combination with opsonizing antibodies was detected only for CD47+/CD20+ cell lines and not for CD47+/CD20‐ cell lines.^46^ Another CD20/CD47 bsAb of the so‐called dual‐variable‐domain immunoglobulin (DVD‐Ig) format also selectively bound to CD20/CD47‐positive cells, even in the presence of a 20‐fold excess of RBCs.^47^ This bsAb did not outperform a combination of CD47 mAb and RTX treatment in terms of inhibition of tumour (Raji) growth in NSG mice, although the impact of the ‘CD47 antigen sink’ was not evaluated, as the bsAb did not cross‐react with mouse CD47.^47^ A phase I clinical trial with this bsAb is recruiting (CTR20192612). A third CD20/CD47 bsAb comprises a CD47 nanobody fused to the C‐terminus of RTX.^48^ This bsAb selectively interacted with CD20/CD47‐positive cells. Here, macrophage‐mediated phagocytosis did not outperform single RTX treatment in vitro, but the bsAb did delay tumour growth of Raji cells significantly better than CD47 or CD20 single block in NOD/Shi‐scid/IL‐2Rγnull(NOG) mice.^48^ Of note, in April 2021, a clinical trial with an analogous approach in which ofatumumab was fused with rhSIRPα (CPO107/JMT601) started recruiting CD20‐positive lymphoma patients (NCT04853329), although no preclinical efficacy or safety data have been published yet. Taken together, the preclinical data with selective targeting of CD47 on B cells clearly provide proof‐of‐concept for B‐cell‐restricted checkpoint inhibition, and results on safety and efficacy in patients are expected in the near future.

### 3.3 Acute myeloid leukaemia

To direct CD47‐mediated blocking towards AML, the antigen CD33 (siglec‐3) has been exploited. CD33 is a transmembrane receptor expressed on cells of the myeloid lineage that is overexpressed on blasts of ∼90% of AML patients.^49^ Both CD33‐targeting antibody drug conjugates and bispecific antibodies were reported to have high affinity for leukaemic blast cells in clinical trials in relapsed AML.^50^, ^51^ A CD47 bsAb comprised of CD33 mAb gemtuzumab and CD47 mAb 11a1 called HMBD004 inhibited CD47‐SIRPα binding, thereby improving phagocytosis of HL60 AML cells and extending survival in a murine xenograft model. Moreover, HMBD004 preferentially bound to CD47+/CD33+ cells in a mixed population with CD47+/CD33‐ cells as measured by flow cytometry.^49^ An analogous approach in which the N‐terminal Ig domain of SIRPα was fused to the variable light chain of a CD33‐targeting IgG1 antibody selectively triggered 70% phagocytosis on the CD33‐positive cell line MOLM‐13 compared to 55% by CD33 mAb alone at a concentration of 10 nM.^52^

Another AML target being evaluated in the context of CD47 bsAbs is CD123 (interleukin‐3 receptor α). CD123 is expressed on AML blasts and AML stem cells, with only moderate expression on normal haematopoietic stem cells.^53^ To exploit CD123 for AML‐selective CD47 blocking, the extracellular domain of SIRPα was fused to a CD123 antibody.^54^ This therapeutic, termed CD123xSIRPα, opsonised the AML cell line MOLM‐13 and augmented antibody‐directed cellular cytotoxicity (ADCC) compared to CD123 mAb (Half maximal effective concentration 10.1 pM vs. 38.5 pM). Furthermore, treatment strongly increased phagocytosis of primary patient‐derived AML cells by autologous macrophages compared to CD123 mAb (90% vs. 20%, respectively). Of note, CD123xSIRPα preferentially bound to MOLM‐13 cells in the presence of a 20‐fold excess of RBCs, highlighting a preferred binding profile that may be associated with low toxicity in humans. Taken together, clear preclinical evidence for AML‐restricted CD47 checkpoint inhibition has been generated for both CD33‐ and CD123‐based targeting. Particularly in view of the DLT of ubiquitous CD47 blocking in AML and MDS, the clinical safety and efficacy of these therapeutics is of clear interest in order to (re)position CD47 blocking for AML.

### 3.4 Solid tumours

CD47 bsAbs have also been developed for several types of solid cancer, including the targeting of mesothelin (MSLN) using a CD47 bsAb of κγ‐body format.^55^ MSLN is a cell surface glycoprotein overexpressed in mesothelioma, gastric, lung, pancreatic, biliary and ovarian carcinoma, as well as childhood AML with limited expression on healthy tissue.^56^ The MSLNxCD47 bsAb augmented phagocytosis of the cell lines NCI‐N87 (30% vs. 50%), HPAC (45% vs. 60%) and OVCAR3 (20% vs. 55%) compared to treatment with parental MSLN mAb.^55^ Compared to MSLN mAb treatment, another MSLNxCD47 bsAb with a high‐affinity MSLN sequence yielded a 4.3‐fold increase in phagocytosis.^57^ Of note, (Fab)’2 fragments of this bispecific also enhanced phagocytosis in combination with an MSLN mAb binding to a different epitope of MSLN compared to MSLN mAb alone, thereby clearly demonstrating the added benefit of blocking CD47 on ADCP in this model.^57^

Another target that is being evaluated for tumour‐directed CD47 blocking is CD70, with CD70 antibodies themselves already being pursued for the treatment of solid cancers.^33^, ^58^ A bsAb combining the variable domain of the CD70 antibody vorsetuzumab with the variable domain of SIRPα‐targeting antibody KWAR23 induced more macrophage‐mediated phagocytosis in four out of four renal carcinoma cell lines compared to treatment with SIRPα‐Fc.^33^ Moreover, treatment of xenografted Burkitt lymphoma cell line Raji with the bsAb strongly inhibited tumour growth in Rag2‐/‐IL2rg‐/‐FSIRPα knock in (SRG) mice, although the treatment effect was similar to the combination of vorsetuzumab and SIRPα mAb.^33^

Finally, the well‐known carcinoma markers epidermal growth factor receptor (EGFR) and human epidermal growth factor receptor 2 (Her‐2) have also been exploited for targeting CD47 therapy. EGFR is a transmembrane receptor tyrosine kinase that promotes tumour cell proliferation, angiogenesis and invasion.^59^ EGFR blocking antibodies have been successfully implemented for the treatment of various types of carcinoma, including breast cancer, renal cell carcinoma, non‐small cell lung cancer (NSCLC) and others.^60^ Using knobs‐into‐holes technology, an EGFR mAb was genetically fused to a rhSIRPα variant that was mutated to have higher binding affinity for CD47 than native rhSIRPα.^61^ In an ELISA‐type assay, the bsAb simultaneously bound to EGFR and CD47. Functionally, EGFRxrhSIRPα increased the phagocytic index of macrophage‐mediated phagocytosis of A341 compared to SIRPα‐Fc single (37% vs. 32%) of xenografted NOD/SCID mice in an ex vivo experimental set‐up. Moreover, EGFRxSIRPα significantly delayed tumour growth of EGFR‐positive A431 cells compared to treatment with EGFR mAb or rhSIRPα. Of note, the engineered SIRPα‐Fc cross‐reacted with CD47 of NOD/SCID mice, thus allowing evaluation of CD47‐related RBC toxicity.^62^ Importantly, EGFRxSIRPα treatment did not impact RBC count, whereas SIRPα‐Fc did reduce RBC count. Another bsAb‐based approach, CD47xEGFR‐IgG1, enhanced EGFR‐directed phagocytosis of cancer cells and promoted cross‐presentation of antigens to engage the adaptive immune system.^63^ Finally, although no preclinical data have been published, a HER2‐SIRPα bispecific mAb‐Trap antibody is being evaluated in a clinical trial for Her2+ cancer patients (NCT05076591) (Figure 3). It has been shown that the combination of trastuzumab and magrolimab overcomes resistance to trastuzumab single treatment and improves phagocytosis in vitro,^64^ providing a clear rationale for this bsAb. Taken together, the tumour antigens MSLN, CD70, EGFR and HER2 are being exploited as targets for target‐selective CD47 blockade for solid cancer with preclinical proof‐of‐concept for enhanced selectivity and activity.

### 3.5 Challenges in tumour‐targeted bispecific antibody‐based CD47 therapy

Although all the above‐described bispecific antibodies and fusion proteins have in vitro therapeutic effects and reduced activity towards RBCs, a challenge of these tumour antigen‐directed approaches is that most of these targets are not truly tumour‐specific but merely overexpressed in cancer. Thus, such bispecific antibodies will also target healthy target antigen‐expressing cell types with high avidity, such as healthy epithelial cells upon EGFR targeting or cardiomyocytes upon Her2 targeting. This might thus unmask additional toxicity issues not previously encountered with CD47‐based therapeutics. Furthermore, targeting CD47 to a particular tumour‐associated antigen carries an inherent risk in often heterogenous cancers. For example, in AML, several leukaemic clones can coexist within one patient, with distinct membrane receptor‐expression profiles.^65^ Furthermore, therapy can change clonality and drive resistance to targeted therapy. Overcoming such heterogeneity in cancer using a CD47‐based bsAb might be achieved by use of an anti‐tag approach, such as targeting of biotin or the fluorescein isothiocyanate (FITC) label, as previously demonstrated by us for targeted activation of tumour necrosis factor receptor (TNFR) superfamily signalling.^66^

## 4 DESIGN OF CD47‐SIRPα BLOCKING IN COMBINATION WITH OTHER THERAPEUTIC STRATEGIES

In addition to the development of novel CD47‐directed tumour‐targeted immunotherapeutics, the implementation of CD47 blocking in combinatorial therapeutic strategies is of particular appeal. Indeed, this appeal is evident from the clinical results upon combined treatment with CD47 antibody and RTX in B‐cell lymphoma and the combination of CD47 blocking with azacytidine in AML and MDS.^67^ In the next section of this review, we will discuss the rationale and available data on combinatorial strategies of CD47 blocking with (1) opsonizing antibodies, (2) systemic therapy and (3) dual innate and adaptive checkpoint immunotherapy.

### 4.1 Combination of CD47‐SIRPα blocking with tumour‐selective opsonizing antibodies

Many of the (pre)clinical studies on CD47‐SIRPα blocking have employed a combination with a tumour‐targeting therapeutic antibody that opsonises cancer cells and triggers FcR‐mediated activation of phagocytes (Figure 2, panel 1). Most prominent among these is the combination with RTX, a CD20‐directed monoclonal antibody that triggers NK‐cell‐mediated ADCC and macrophage, monocyte, neutrophil and dendritic cell‐mediated ADCP of CD20‐positive cells.^68^, ^69^ Indeed, in the first published clinical trial, the combination of CD47 mAb with RTX triggered a 40% ORR and 33% CR in DLBCL and 71% ORR and 43% CR in follicular lymphoma.^18^ These clinical effects are in agreement with various preclinical studies. For example, the combination of the CD47 mAb B6H12 with RTX synergistically enhanced phagocytosis of primary NHL compared to RTX and CD47 single treatment.^70^ Furthermore, the combination of the CD47 mAb magrolimab eliminated disease in three of four Raji‐engrafted NSG mice, whereas monotherapy with either mAb only delayed tumour growth.^71^ Similarly, SIRPα mAbs and rhSIRPα increase phagocytosis of RTX opsonised tumour cells in vitro and in vivo.^33^, ^72^ Finally, a macrocyclic peptide D4‐2 that binds to SIRPα to competitively block CD47 interaction increased phagocytosis of RTX opsonised lymphoma cells by bone marrow‐derived macrophages (BMDM) by 50%.^73^ Treatment with D4‐2 alone did not trigger phagocytosis. Thus, the combination of CD47 blocking with RTX is a prominent and effective strategy that warrants further exploration in clinical trials.

Similarly, the combination of the standard‐of‐care Her2‐targeting antibody trastuzumab with CD47‐SIRPα blocking improved breast cancer phagocytosis.^73^, ^74^, ^75^ For example, the above‐described macrocyclic peptide D4‐2 increased phagocytosis of breast cancer by BMDM by ∼10% compared to trastuzumab treatment alone.^73^ CD47 blocking also augmented the in vitro ADCP of Her2+ breast cancer cell lines.^74^ In xenograft mouse studies where Her2+ breast cancer cells were injected and treated with trastuzumab and CD47 blocking antibody, survival time was increased.^74^, ^75^ Similarly, the combination of SIRPα mAb KWAR23 with trastuzumab significantly increased macrophage phagocytosis of the SK‐BR‐3 Her2+ breast cancer cell line from 25% to 90%^33^ compared to trastuzumab alone.^33^ Currently, the combination of trastuzumab and a rhSIRPα‐Fc fusion protein (ALX148) is being evaluated in a phase 1 clinical trial (NCT03013218).

In a similar fashion, the targeting of carcinoma‐expressed EGFR using cetuximab and SIRPα mAb KWAR23 significantly enhanced macrophage phagocytosis of colon adenocarcinoma cell‐line (DLD‐1) from 35% to 90%.^33^ Furthermore, the combination of cetuximab with magrolimab has been evaluated preclinically (data not presented) and, thereafter, evaluated in a phase 1b/2 clinical trial. In this trial, 45% of relapsed or refractory mKRAS colorectal patients experienced SD, with a median progression free survival of 1.9 months and a median overall survival of 10.4 months.^16^

Importantly, the outcome of these combinations depends on the tumour type and the immune microenvironment. In this respect, we previously identified that CD47 expression only impacted survival in a subtype of DLBCL patients, specifically non‐Germinal Centre B‐cell (GCB) (or activated B cell [ABC] plus unclassified) DLBCL patients.^6^ Moreover, only non‐GCB cell lines responded to CD47 blocking in vitro. These findings are in line with the clinical results of magrolimab combination with RTX in DLBCL patients, where 67% of ABC‐DLBCL patients responded and only 17% of GCB‐DLBCL patients responded.^18^ Taken together, these two studies indicate that ABC‐DLBCL might benefit more from the combination of CD47 with RTX, although studies in larger patient cohorts are required to confirm this hypothesis. Such differences likely exist for other cancer types, for example, microsatellite instable (MSI) cancers, with a clear need to identify optimal combinatorial strategies as well as predictive biomarkers. The reason(s) underlying such differential responses to CD47 blocking may be related to other ‘don't eat me’ and ‘eat me’ signals in the tumour microenvironment (TME). For example, CD47 blocking can be impacted by various other anti‐phagocytic signals (leukocyte immunoglobulin like receptor B1 [LILRB1],^76^ programmed cell death protein [PD]‐1/PD‐ligand [L]1^77^, ^78^) and pro‐phagocytic signals such as PS,^79^ calreticulin^80^, as well as signalling lymphocytic activation molecule family member 7 (SLAMF7),^81^ although we previously identified that SLAMF7 expression was not required for effective treatment with CD47 blocking in DLBCL.^11^ Furthermore, distinct infiltration patterns of macrophages and other innate immune cells as well as T cells may dictate the response.

### 4.2 Combining CD47‐SIRPα blocking with systemic therapy

Another strategy being explored for CD47 blocking therapeutics is the combination with chemotherapeutics that kill rapidly proliferating cancerous cells (Figure 2, panel 3). In the process of dying, a host of ‘eat me’ signals, including but not limited to phosphatidylserine, calreticulin and HMGB1, are upregulated on the surface of cancer cells or secreted to drive phagocytic removal of dying cells.^82^ Thus, the combination of chemotherapeutic drugs with CD47 blocking might help further shift the balance towards phagocytic removal of cancer cells. The added value of combination treatment of CD47 blocking with chemotherapy has, for instance, been demonstrated in different cancer models for the chemotherapeutic drug doxorubicin.^83^, ^84^ Combination of doxorubicin with a CD47 mAb in a 4T1 breast cancer model significantly reduced tumour growth through macrophage‐mediated ADCC.^85^ Secondly, phagocytosis of the osteosarcoma cell line (MNNG/HOS) by BMDM differentiated into macrophages was increased by 20% upon treatment with doxorubicin and CD47 mAb compared to either CD47 mAb or doxorubicin treatment alone.^86^ Thirdly, treatment of MC38 xenografts with doxorubicin 1 day prior to treatment with a PD‐L1‐SIRPα fusion protein significantly delayed tumour growth compared to either monotherapy.^83^ In all these studies, the ‘eat me’ signal calreticulin was upregulated on the cell surface upon doxorubicin treatment, suggesting that a shift in the phagocytic balance can increase phagocytic removal.

A second class of drugs that have been evaluated in combination with CD47 blocking are epigenetic drugs such as azacytidine, which inhibit aberrant DNA methylation that occurs in cancer.^87^ Similar to chemotherapeutics, azacytidine triggered the upregulation of ‘eat me’ signals. However, Treg and innate immune infiltration were also upregulated in a PDAC tumour model, suggesting diverse mechanisms.^88^ Treatment with the demethylating agent azacytidine alone is associated with good clinical outcome and a 50% ORR in high‐risk MDS.^89^ In an ongoing phase 1b clinical trial, combination treatment with azacytidine and CD47 mAb increased the ORR in high‐risk MDS patients to 92%, with 12 patients (50%) achieving CR. Furthermore, in preclinical studies, the combination of CD47 mAb and azacytidine significantly enhanced phagocytic elimination of AML cells by human macrophages in vitro, enhanced clearance of AML in vivo, and prolonged survival compared to single agent treatment.^88^ In AML patients, this treatment induced an ORR of 64%, with nine patients achieving CR (41%).^90^ Currently, an expansion cohort is ongoing (NCT03248479).^17^ Furthermore, the antibodies ALX148 and AK117 are also being evaluated in combination with azacytidine in clinical trials (NCT04417517, NCT04900350).

A third class of drugs being tested in combination with CD47 blocking are proteasome inhibitors, which trigger cytotoxic elimination of cancer cells. This class of drugs is used in the treatment of multiple myeloma (MM) and mantel cell lymphoma (MCL) and is being evaluated for various other cancer types. Proteasome inhibitors such as bortezomib, carfilzomib and ixazomib also upregulate ‘eat me’ signals such as galectin 3, galectin 9, HSP90 and calreticulin. It is therefore not surprising that the combination of proteasome inhibition and TTI‐621 potentiated phagocytic uptake in vitro and significantly delayed MM tumour growth compared to single agent treatment in a mouse model.^91^ This strategy is being evaluated in clinical trials with relapsed MM patients, where TTI‐622 is being tested in combination with carfilzomib plus dexamethasone (NCT03530683) and antibody AO‐176 is being tested in combination with bortezomib/dexamethasone (NCT04445701).

Interestingly, accumulating evidence points to CD47‐SIRPα blocking antibodies not only triggering phagocytosis but also protecting cells from death by protective autophagy.^92^ The induction of protective autophagy upon CD47 treatment starts with upregulation of LC3, BECN1, ATG5 and ATG7, which has a cytoprotective role in malignant cells.^93^ Indeed, treatment with a CD47 blocking antibody in NSCLC triggered cytoprotective autophagy. Subsequent combination of CD47 antibody with the autophagy inhibitor chloroquine improved macrophage‐mediated phagocytosis of NSCLC cells from 30% (rhSIRPα) to 50% (chloroquine + rhSIRPα)^94^ (Figure 2, panel 4). Similarly, rhSIRPα treatment of glioblastoma cancer cells upregulated autophagy markers, such as LC3 and SQSTM1. Moreover, combination treatment with rhSIRPα and chloroquine again significantly enhanced phagocytosis from 30% (rhSIRPα) to 45% (rhSIRPα + chloroquine). Cotreatment also significantly extended the median survival of glioblastoma tumour‐bearing mice compared to single rhSIRPα treatment from 38 to 49 days.^95^ Notably, the anti‐glioblastoma effect of disrupting CD47‐SIRPα signalling was at least partly caused by additional CD8+ T cells, as the effect was diminished by depletion of CD8+ T cells.

For any of the described combinations, the timing and dosing regimen should be carefully calibrated. In this respect, when mice were treated with chemotherapeutic agents (cyclophosphamide and paclitaxel) after CD47 therapy, the CD8+ T‐cell response in vivo was abrogated upon rechallenge of mice with the same tumour cell line. In contrast, when chemotherapeutics were given 1 day before CD47 blocking, CD47 not only synergised with chemotherapy for tumour control but also preserved the host memory response against relapsing tumours.^96^ In addition, certain therapeutics can upregulate CD47 expression, as described before for chemotherapeutics in triple‐negative breast cancer,^97^ but also anti‐angiogenic therapy (anti‐vascular endothelial growth factor [VEGF]) in NSCLC.^98^ Most likely, CD47 expression is used as an escape mechanism by tumour cells that remain after chemotherapy. Thus, the impact and timing of (chemo)therapeutic treatment on CD47 expression should be monitored, and consequently, the timing of CD47 blocking could be tailored for optimal activity in future clinical trials. In this respect, it would also be of interest to investigate whether such drug‐induced upregulation of CD47 expression correlates with improved CD47 blocking therapeutic effects.

## 5 DUAL TARGETING OF INNATE (CD47‐SIRPα) AND ADAPTIVE IMMUNITY

The efficacy of CD47 blocking in several mouse models relied on T‐cell and NK‐cell responses, with knockout of these cells negating therapeutic activity.^83^, ^99^ Moreover, in cutaneous T‐cell lymphoma, treatment with intratumoural rhSIRPα injections triggered expansion of NK and CD8+ T cells in the TME.^100^ Similarly, in mice xenografted with DLD1‐cOVA‐RFP+ cancer cells and treated with CD47 blocking mAb, the popliteal lymph nodes contained more proliferating OT‐I T cells in comparison to IgG treatment. Moreover, these macrophages primed an antitumour CD8+ T‐cell response in vivo that protected mice from rechallenge with the same cancer cell line.^96^

Thus, the combination of CD47‐SIRPα with further activation of adaptive T‐cell immunity may yield a more effective strategy to induce durable antitumour activity^78^, ^101^ (Figure 2, panel 2). In this respect, the PD‐1/PD‐L1 checkpoint axis for T cells is an interesting target with proven clinical activity in many cancers (as reviewed in Ref.^62^). In immunocompetent C57BL/6 mice, xenografted MC38 colon cancer cells treated with PD‐L1 mAb and SIRPα‐Fc significantly reduced tumour growth compared to monotherapy.^83^ Interestingly, in the absence of CD8+ T cells, the effect of PD‐L1 mAb and rhSIRPα on tumour growth was completely abrogated.^83^ Furthermore, whereas in a poorly immunogenic B16F10 syngeneic melanoma mouse model CD47 blocking did not synergise with the B16F10‐specific monoclonal antibody TA99 (anti‐TRP‐1), combination with PD‐L1 blocking did control tumour growth in vivo and triple combination with anti‐TRP‐1 cured a majority of mice (60%).^83^

A second combinatorial strategy to reactivate innate and adaptive immunity that has been evaluated preclinically is the combination of CD47 blocking with cytotoxic T‐lymphocyte associated protein 4 (CTLA‐4) blocking antibodies. CTLA‐4 is an important immune checkpoint on T cells that competes with CD28 for B7‐1/2 binding to competitively inhibit early T‐cell activation.^102^ In a study with oesophageal cell carcinoma, treatment with CD47 blocking increased the expression of both CTLA‐4 and PD‐1.^87^ Correspondingly, the combination of CD47 blocking with CTLA‐4 mAb significantly inhibited mouse xenografted tumour growth compared to CD47 single treatment.^103^ Similar effects were observed in C57BL/6 mice treated with Panc02 (pancreatic cancer cell line), where CD47 mAb in combination with CTLA‐4 treatment inhibited tumour growth more profoundly than single treatment.^86^ Thus, preclinical evidence provides clear proof‐of‐concept for combining PD‐1/PD‐L1 or CTLA‐4 checkpoint inhibition with CD47 blocking.

### 5.1 CD47‐based therapeutics with dual immunomodulatory activity

To further exploit this dual immunomodulatory strategy, bsAbs that target both adaptive and innate immunity have been developed (Figure 2, panels 2 and 5). An example of such dual targeting of innate and T‐cell‐mediated immunity is an engineered CD47‐targeting PD‐L1 bsAb (IBI322) (Figure 3). This bsAb has a 30‐fold lower affinity for CD47 than the parental CD47 mAb. Consequently, IBI322 uptake was increased in PD‐L1‐CD47‐positive tumours and decreased in blood depot organs compared to the parental CD47 mAb. IBI322 had a favourable toxicity profile in nonhuman primates (NHPs), with much milder adverse effects on RBCs than in‐house generated magrolimab. However, as the binding affinity of IBI322 to cynomolgus CD47 was not disclosed, potential toxicity concerns in humans remain unanswered. IBI322 treatment outperformed treatment with a combination of PD‐L1 + CD47 mAb in controlling tumour growth of PDL1^+^CD47^+^ Raji cells after injection with human PBMC.^104^ Currently, IBI322 is being evaluated in clinical trials as monotherapy for solid cancers and haematological diseases (NCT04912466, NCT04338659, NCT04328831, NCT04795128). The first clinical results were presented on the site of innovent as 20% ORR (according to RECISTv1.1) and 74.1% treatment‐related AE that was mostly grade 1–2 anaemia. HX‐009 is another PD‐1/CD47 bispecific approach, in this case comprising a PD‐1 antibody fused to soluble SIRPα, which is currently being evaluated in a clinical trial (NCT04097769). Although preclinical data have not been presented, the first clinical trial with HX‐009 treatment of 21 patients with solid cancer was associated with manageable grade 1 or 2 toxicity in 46% of patients. Furthermore, of the 18 evaluable patients, three experienced a PR and six experienced SD.^105^

In addition to targeted inhibition of negative signals, the activation of costimulatory receptors on T cells is of interest. For instance, we have developed a fusion protein comprising the extracellular domain of SIRPα fused to soluble 4‐1BBL, termed DSP‐107.^72^ The 4‐1BBL domain targets T cells expressing the costimulatory receptor 4‐1BB, a well‐established surrogate marker for tumour‐reactive T cells, and triggers the activation of cytotoxic T lymphocytes. Single treatment with DSP‐107 or in combination with RTX triggered significant phagocytosis of a panel of DLBCL cancer cell lines. Moreover, activation of 4‐1BB costimulatory signalling triggered augmented T‐cell cytotoxicity in vitro in an effector‐to‐target ratio‐dependent manner only upon CD47‐specific binding of DSP‐107.^106^ DSP‐107 is currently being evaluated in a phase I clinical trial in NSCLC and advanced solid tumours (NCT04440735). In an analogous approach, the bifunctional fusion protein SL‐172154 is designed to simultaneously inhibit CD47/SIRPα signalling and activate the costimulatory receptor CD40 on antigen presenting cells (APCs). CD40 signalling presents a crucial licensing step for antigen presentation by APCs and subsequent CD8+ and CD4+ T‐cell activation. Interestingly, although macrophages were more potent in the uptake of cancer cells, the cross‐priming of CD8+ T cells mediated by type I interferon (IFN) largely depends on dendritic cells.^96^, ^106^, ^107^ In BALB/C mice, murine SIRPα‐Fc‐CD40L increased tumour rejection rates and long‐term immunity compared to treatment with CD47 mAb, CD40 agonist antibody, or the combination of these.^108^ Two phase I clinical trials with SL‐172154 are ongoing in patients with gynaecological cancers (NCT04406623) or squamous cell carcinoma of the skin and head and neck (NCT04502888).

Thus, T cells play an integral role in the efficacy of CD47 blocking preclinically, and bsAbs that cotarget T cells and macrophages have good preclinical activity and safety. It will be important to determine whether and how T cells are involved in the therapeutic effect of CD47 blocking in humans, for example, by defining the T‐cell receptor repertoire after treatment and screening for the frequency of tumour reactivity within the T‐cell population. If T‐cell activation proves important in humans, further combinations tailored towards synergizing innate and adaptive immunity, such as the DSP107 and SL‐172154 fusion proteins described above, may yield significant advances in therapeutic outcomes.

## 6 CONCLUSIONS

In this review, the current state of CD47 blocking in the clinic and associated limitations, as well as strategies to overcome those limitations, such as circumventing RBC toxicity and increasing the efficacy of CD47‐SIRPα blocking are discussed, including next generations of monoclonal antibodies and bifunctional therapeutics. Additionally, the rationale and supporting evidence for various types of combinatorial strategies that could synergise with CD47 blocking treatment were reviewed.

Although the use of priming and maintenance dose of magrolimab helped reduce anaemia as observed in the first clinical trials, the recent halt on the magrolimab ENHANCE trials in AML/MDS patients highlights the pivotal need to develop strategies that further mitigate toxicity in order to unlock the immune (re)activation potential of CD47‐SIRPα therapy. In this respect, circumventing toxicity using novel antibody formats that have been designed to target CD47 on a distinct epitope that enables a unique RBC sparing property is of particular promise. The first dose‐escalation studies with this type of antibody, lemzoparlimab and AK117, reached doses of 10 and 20 mg/kg without DLT. In contrast, conventional therapeutics such as SRF231, TTI‐621 and TTI‐622 reach MTDs of 4, 0.2 and 8 mg/kg, respectively. Encouragingly, lemzoparlimab given at 20–30 mg/kg with RTX triggered 57% ORR in refractory/relapsed NHL patients who were heavily pretreated with RTX.^29^ Similar effects were obtained with the first clinical trial with BI 765063 (SIRPα mAb), which gave clinical benefit in 45% of the patients with solid tumours with moderate AEs.^36^ The combination of such next‐generation antibodies with opsonizing mAbs such as RTX, cetuximab and trastuzumab holds clear promise. Similarly, early combination studies of azacytidine and magrolimab gave good clinical responses in MDS and AML patients. Thus, overcoming the apparent toxicity of this combination with next‐generation mAbs is anticipated to yield superior safety and possibly efficacy.

The use of bispecific tumour‐targeted antibodies is another promising approach to not only increase tumour‐selective activity but also to reduce possible RBC toxicity. However, with few exceptions, such as EGFR variant III and under‐glycosylated Mucin‐1, the tumour targets in such constructs are not uniquely expressed on cancerous cells and are also found on healthy cell types. Consequently, treatment with such bispecific antibodies may unmask on‐target toxicity. Furthermore, under the selection pressure of treatment, clonal drift may occur, leading to resistance to targeted therapy and target antigen‐negative relapse.

To push the clinical application of CD47 blockade forward, it will be important to evaluate the impact of T cells and dendritic cells on the therapeutic effect of CD47‐SIRPα blockade with analyses of T‐cell clonality in the blood and TME of patients. Indeed, since T cells were required in several murine models for therapeutic efficacy, further focus on combinatorial innate/adaptive immune targeting appears warranted. Recently, such a dual immunomodulatory strategy was evaluated preclinically, where CAR‐T cells secrete SIRPα‐Fc to enhance phagocytosis and therapeutic effects of CAR‐T cells in solid tumours.^109^ Additionally, the timing of CD47 mAb in combination with chemotherapy impacts the CD8+ T‐cell response in vivo and requires further investigation. Moreover, since the expression of CD47 itself can be modulated in certain types of cancer upon therapy, this modulation of expression may offer opportunities for optimal combinatorial therapy design.

In conclusion, although CD47‐SIRPα blocking has several challenges in clinical application, prominent clinical activity has been observed. Indeed, the advent of novel monoclonal/bsAb formats and combination strategies point towards the clear potential for increased tumour selectivity and efficacy and reduced toxicity of CD47‐SIRPα‐based immunotherapy.
